# Copper sensing transcription factor ArsR2 regulates VjbR to sustain virulence in *Brucella abortus*

**DOI:** 10.1080/22221751.2024.2406274

**Published:** 2024-09-19

**Authors:** Feijie Zhi, Kemeng Liu, Hao Geng, Mengru Su, Jian Xu, Lei Fu, Ke Ma, Pengcheng Gao, Lvfeng Yuan, YueFeng Chu

**Affiliations:** a State Key Laboratory for Animal Disease Control and Prevention, College of Veterinary Medicine, Lanzhou Veterinary Research Institute, Lanzhou University, Chinese Academy of Agricultural Sciences, Lanzhou, People’s Republic of China; b Gansu Province Research Center for Basic Disciplines of Pathogen Biology, Lanzhou, People’s Republic of China; c Key Laboratory of Veterinary Etiological Biology, Key Laboratory of Ruminant Disease Prevention and Control (West), Ministry of Agricultural and Rural Affairs, Lanzhou, People’s Republic of China

**Keywords:** *Brucella*, ArsR, T4SS, VjbR, virulence

## Abstract

Brucellosis, caused by the intracellular pathogen *Brucella*, is a major zoonotic infection that promotes reproductive disease in domestic animals and chronic debilitating conditions in humans. The ArsR family of transcriptional regulators plays key roles in diverse cellular processes, including metal ion homeostasis, responding to adverse conditions, and virulence. However, little is known about the function of ArsR family members in *Brucella*. Here, we identified ArsR2 as a nonclassical member of the family that lacks autoregulatory function, but which nevertheless plays a vital role in maintaining copper homeostasis in *B. abortus*. ArsR2 is a global regulator of 241 genes, including those involved in the VirB type IV secretion system (T4SS). Significantly, ArsR2 regulates T4SS production in *B. abortus* by targeting VjbR which encodes a LuxR-type family transcriptional regulator. Moreover, copper modulates transcriptional activity of ArsR2, but not of VjbR. Furthermore, deletion of *arsR2* attenuated virulence in a mouse model. Collectively, these findings enhance understanding of the mechanism by which ArsR proteins regulate virulence gene expression in pathogenic *Brucella* species.

## Introduction

Brucellosis, caused by bacteria of the *Brucella* genus, is a contagious zoonotic infectious disease. Brucellosis induces reproductive diseases that mainly are characterized by abortion and infertility in domestic animal hosts, whereas human disease triggers chronic debilitating conditions that frequently involve recurrent fever and incapacitating musculoskeletal damage [1]. *Brucella* predominantly survives intracellularly in natural hosts, and must respond to the intracellular environment and subvert the host immune response to facilitate proliferation in macrophages [2,3].

The type IV secretion system (T4SS) in *Brucella* is a crucial virulence factor that comprises 12 proteins [4,5]. T4SS plays a key role in controlling trafficking of *Brucella*-containing vacuoles (BCVs) to avoid bacterial killing and degradation in phagolysosomes [6,7]. Biogenesis of replication-permissive organelles derived from host endoplasmic reticulum (rBCVs) relies on T4SS as various *virB* mutant strains are incapable of forming these organelles which leads to stalling in endosome-like *Brucella*-containing vacuoles (eBCVs) and eventual death [8,9]; Moreover, formation of autophagy-related vacuoles (aBCVs) and bacterial degradation also depend on T4SS [10]. In addition, the complex is crucial for inducing an immune response by translocation of bacterial effectors, including VceC, into host cells which influences production of inflammatory cytokine IL-6 [11–14]. Nevertheless, the regulatory mechanisms that underpin T4SS in *Brucella* remain unclear.

Bacteria have evolved diverse signal transduction systems, including factors that control gene expression at the transcriptional level, that enable adaptation to an array of environmental changes. *Brucella* contains multiple transcription factor families, such as GntR, LysR and so on, which are involved in virulence [15]. The arsenic acid-resistant (ArsR) family of transcriptional regulators are distributed widely among bacteria. The first member of the ArsR family was identified in *Escherichia coli*, which can repress the transcription of the *ars* operon and participate in sequestering excess of heavy metal ions [15]. The N-terminus of ArsR possesses a winged helix-turn-helix DNA-binding domain that is responsible for binding of target promoters [16]. ArsR family proteins also are involved in maintenance of metal ion homeostasis under extreme environmental conditions, including the presence of heavy metal ions and superfluous biologically-required metal ions [17,18]. Furthermore, ArsR regulators play key roles in numerous important cellular processes, including biofilm formation, primary and secondary metabolism, responses to adverse conditions, and virulence [15]. However, the relationship between these regulators and metal ion homeostasis and bacterial virulence remains largely unknown in *Brucella*.

*Brucella abortus* is a pathogen of diverse animals, including cattle and sheep, and also is a human pathogen [3]. *B. abortus* S2308 is a standard strain for studying virulence, whereas strain A19 is utilized as a vaccine for immunizing cattle [19,20]. However, *B. abortus* A19 exhibits certain shortcomings as a vaccine, including residual virulence [21,22]. ArsR2 was encoded by *BAB2_0334* and *DR186_RS12705* in *B. abortus* S2308 and A19, respectively. Here, we identified an atypical ArsR-like transcriptional factor (ArsR2) in both S2308 and A19 that maintains copper homeostasis and regulates the vital T4SS virulence factor through VjbR, a LuxR-type family transcriptional regulator. Copper ions inhibit binding of ArsR2 to the *vjbR* promoter thereby activating transcription of the gene. In addition, mouse infection studies revealed that ArsR2 is an important virulence factor in *B. abortus*. Our results led to the conclusion that ArsR2 is a copper sensing transcription factor in *B. abortus*, and controls virulence by regulating T4SS production by VjbR.

## Results

### ArsR2 in *Brucella* is an atypical member of the ArsR family

**Figure 1.**
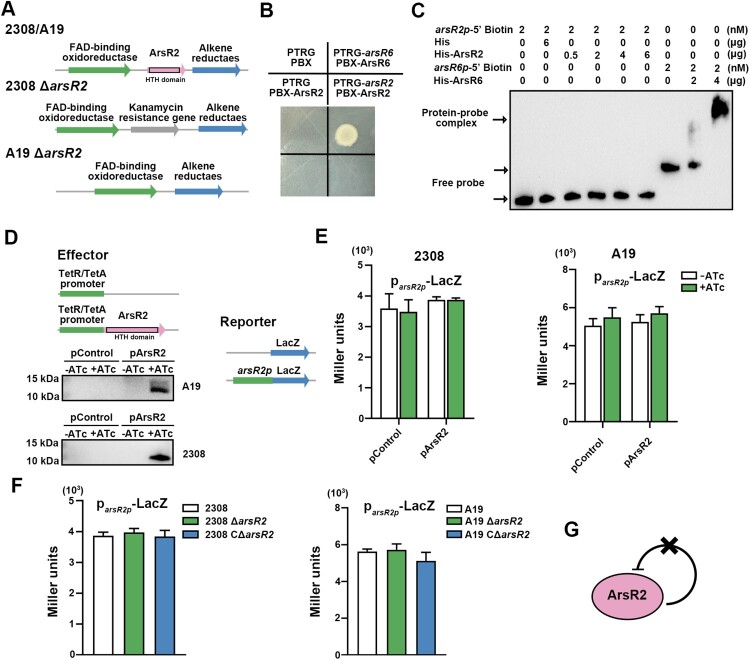
ArsR2 lacks self-regulatory properties. (A) Schematic representation of *B. abortus* S2308 and A19 Δ*arsR2* and complemented strains. (B) B1H assays to probe the interaction between ArsR2 and cognate promoter. E. coli XR reporter strains containing the pTRG and pBX plasmids and derivatives were cultured and spotted on LB plates in the presence or absence of streptomycin and 3-AT. The pTRG and pBX empty vectors were used as negative controls. pTRG-ArsR6 and pBX-ArsR6 were positive controls. Plasmids pBRG and pBX-ArsR2 were used as self-activation controls. (C) EMSA of His-ArsR2 and the *arsR2* promoter fragment. His-tag was incubated with the *arsR2* promoter as a negative control. ArsR6 was incubated with *arsR6p* as a positive control as it previously was shown that ArsR6 bound to this promoter [25]. (D) Schematic diagrams of effector, reporter, and control constructs used in *lacZ* reporter assays. Western blot of ArsR2-flag levels in *B. abortus* S2308 and A19 strains containing the pBB-PZT1-ArsR2 plasmid to induce ArsR2 protein expression with 200 ng/mL ATc. The *arsR2p* fragment comprises approximately 500-bp upstream of the *arsR2* start codon. (E) Activity of a plasmid-borne p*arsR2p*-*lacZ* transcriptional reporter in strains S2308 and A19 with or without ATc. The pControl and p*arsR2p*-*lacZ* plasmids or pArsR2 and p*arsR2p*-LacZ were electroporated into *B. abortus* S2308 or A19, respectively. The β-galactosidase activity was detected in the presence or absence of 200 ng/mL ATc. pControl and pArsR2 indicate pBB-PZT1 empty vector and pBB-PZT1-ArsR2, respectively. Strains were grown in TSB to OD_600_ 0.6 in the absence or presence of 200 ng/mL ATc. (F) Activity of p*arsR2p*-*lacZ* transcriptional reporter in *B. abortus* S2308 or A19 and derivatives. The p*arsR2p*-*lacZ* plasmid was electroporated into the required strains and β-galactosidase activity was assayed in strains grown in TSB to OD_600_ 0.6. (G) Model for lack of ArsR2 autoregulation.

ArsR transcription factors are distributed broadly and are well-conserved in prokaryotes. ArsR regulators, including ArsR2 in *Brucella*, possess a helix-turn-helix domain that mediates binding to promoter regions and heavy metal ions (Figure 1(A)). Sequences of ArsR2 homologs in different *Brucella* species were compared, and secondary structures were predicted and visualized by Clustal Omega and ESPript 3.0, respectively. ArsR2 proteins are highly conserved in *Brucella* (Figure S1(B,C)). The unrooted phylogenetic tree revealed that ArsR2 is most closely related to ArsR transcriptional regulators of *Pseudomonas* (Figure S1(D)). Strains with *arsR2* deletions (Δ*arsR2*) were constructed to probe the physiological functions of the protein in *B. abortus* (Figure 1(A)). PCR confirmed deletions in both 2308-Δ*arsR2* and A19-Δ*arsR2* (Figure S2). In contrast, Western blot revealed the presence of ArsR2 protein in detergent extracts of the complemented strains 2308 CΔ*arsR2* and A19 CΔ*arsR2* (Figure S2).

ArsR family members, e.g. AcsR in *Acidithiobacillus caldus* [23], autoregulate expression by binding to the cognate promoters. ChIP-seq was performed to explore whether ArsR2 also recognizes the cognate promoter. However, the *arsR2* promoter region was not significantly enriched in ChIP-seq which suggests that ArsR2 lacks an autoregulatory role in *B. abortus*. Bacterial one-hybrid assays (B1H) verified that ArsR2 does not bind the cognate promoter region (Figure 1(B)). We used electrophoretic mobility shift assays (EMSA) to show that ArsR2 does not bind to its own promoter (Figure S3(A)). No complex was detectable with ArsR2 and the promoter region, although the ArsR6 homolog formed a distinct complex with the *arsR6* promoter (Figure 1(C)) which further indicates that ArsR2 does not interact directly with the *arsR2* promoter region. We next assessed whether ArsR2 autoregulates expression in *B. abortus*. Anhydrotetracycline (ATc) control of plasmid-based ArsR2 expression in *B. abortus* demonstrated production of ArsR2 by the inducer (Figure 1(D)). However, ATc-mediated overexpression of ArsR2 did not alter activity of an *arsR2-lacZ* transcriptional fusion (Figure 1(E)). Similarly, no significant differences were evident in reporter activity in *B. abortus* and its derivatives (Figure 1(F)). Therefore, multiple lines of evidence revealed that ArsR2 in *B. abortus* is not a classical member of the ArsR family as the protein lacks autoregulatory activity (Figure 1(G)).

### ArsR2 possesses higher copper chelating activity

**Figure 2.**
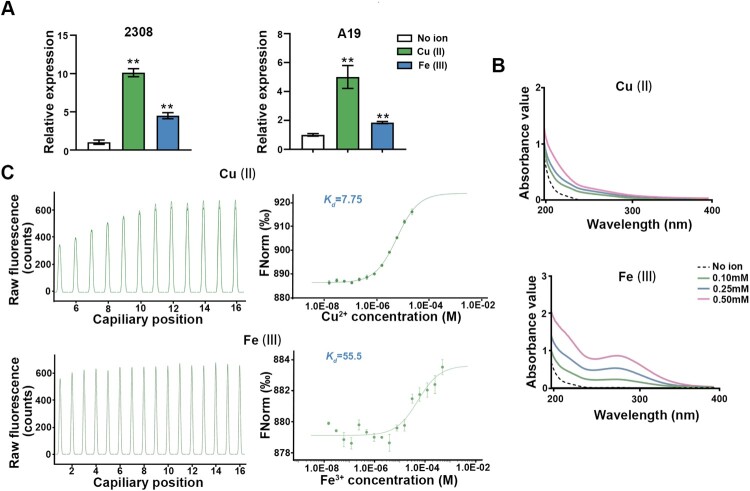
ArsR2 is a metal-binding protein. (A) qRT-PCR of *arsR2* transcript levels in *Brucella* S2308 and A19 in the presence or absence of Cu (II) or Fe (III). Bacteria were grown in TSB medium to OD_600_ 0.6 and incubated with metal ions for 1 h before RNA isolation. Data represent mean and standard deviation. **<0.01 by unpaired two-tailed t tests with Welch’s correction. (B) Absorption spectra of ArsR2 (50 μg/mL) after incubation in different concentrations of Cu (II) or Fe (III). (C) Interactions between ArsR2 and Cu (II) and Fe (III) analyzed by MST. *K_d_* represents the dissociation constant.

As a major function of ArsR family members is maintenance of metal ion homeostasis under extreme environmental conditions, we explored whether ArsR2 binds metal ions. First, qRT-PCR revealed that expression of *arsR2* increased in the presence of copper or ferric ions (Figure 2(A)) indicating that gene expression is modulated by metal ions. Based on this, we asked if ArsR2 directly binds to metal ions. Second, the tag was cleaved from His-tagged ArsR2 using enterokinase (Figure S3(B)) and ArsR2 binding to metal ions was monitored by absorption spectrum changes in the ultraviolet region. Absorbance values increased significantly in the presence of copper or ferric ions (Figure 2(B) and Figure S3(C)) which indicates that ArsR2 can specifically bind copper or ferric ions. Microscale thermophoresis (MST) verified that ArsR2 bound to Cu (II) and Fe (III) (Figure 2(C)). The apparent dissociation constants (*K_d_*) of ArsR2 bound to Cu (II) and Fe (III) were 7.7 μM and 55 μM, respectively, indicating that ArsR2 has a higher affinity for the former. Together, these findings demonstrate that ArsR2 is a metalloregulatory protein prompted further characterization of the role of the protein in metal ion homeostasis.

### Deletion of *arsR2* enhances sensitivity to copper

**Figure 3.**
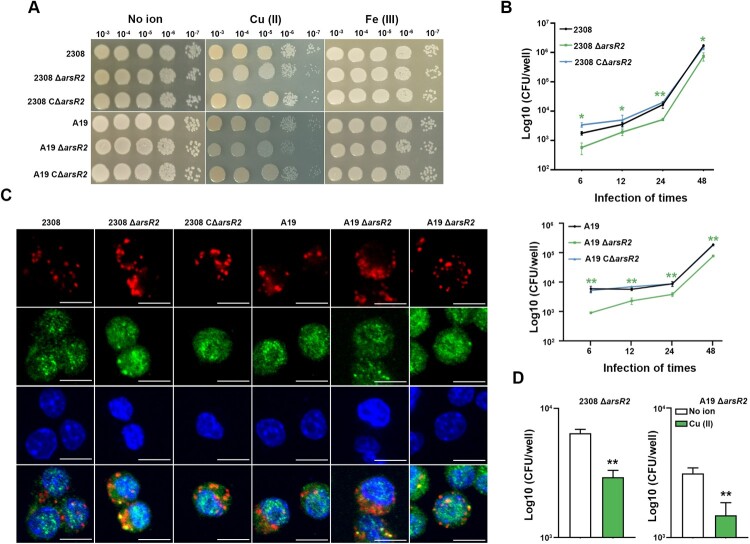
ArsR2 response to copper toxicity. (A) Growth of *Brucella* and its derivatives in TSB medium supplemented with copper or ferric ions. Bacteria were grown in TSB to OD_600_ 0.6. Bacteria were diluted 100-fold in TSB at 37°C with continuous shaking for 72 h. OD_600_ was measured at different time points. (B) Ten-fold serial dilutions of *Brucella* and derivatives were grown to OD_600_ 0.6 and spotted on TSA containing 2 mM copper or 1 mM ferric ion. (C) Intracellular replication of *Brucella* and deletion derivatives. Data represent mean and standard deviation of *N* = 3 (independent biological replicates). *<0.05 by one-way analysis of variation followed by Tukey’s multiple-comparison test. (D) Representative confocal micrographs of rBCVs formation in RAW264.7 infected with *Brucella* or deletion derivatives (red) for 12 h and labelled for the LAMP1 marker protein (green). eBCVs is LAMP1-positive and rBCVs is LAMP1-negative. Results are representative of at least three independent experiments. Scale bar, 10 µm. (E) Bacterial intracellular survival was detected at 24 h post-infection supplemented with 200 μM copper. Data represent mean and standard deviation. *<0.05 by unpaired two-tailed t tests with Welch’s correction.

The identification of ArsR2 as a metalloregulatory protein prompted further characterization of its role in metal ion homeostasis. Growth of wild-type and Δ*arsR2* was indistinguishable at 37°C in TSA plates which indicates that ArsR2 is not essential in *B.abortus* in rich culture conditions (Figure 3(A)). However, growth of Δ*arsR2* strains was reduced in the presence of copper ions, but not in the presence of ferric ions, compared to the parental strains and was fully restored in the CΔ*arsR2* (Figure 3(A)). Thus, the preceding data demonstrate that ArsR2 is required for copper resistance in *B. abortus*. Although ArsR2 is not required for growth in rich culture medium, we examined whether the protein affects intracellular survival of *B. abortus*. The intracellular load of the Δ*arsR2* was reduced significantly in RAW264.7 murine macrophages compared to the parental strain, but was restored fully in CΔ*arsR2*, which suggests that deletion of the gene impairs intracellular survival of *B. abortus* (Figure 3(B)). *Brucella* replication in rBCVs has been described for multiple cell types, including RAW264.7 [10,24]. Therefore, we observed the formation of rBCVs by immunofluorescence which showed that deletion of *arsR2* impaired rBCVs production (Figure 3(C)). We observed previously that copper ions reduce intracellular replication of *B. suis* S2 [25]. As ArsR2 binds copper ions, we assessed whether the protein affects intracellular survival of *B. abortus* in the presence of these ions. Intracellular replication of Δ*arsR2*, was inhibited by exogenous copper (Figure 3(D)). In conclusion, the results show that ArsR2 is required for intracellular survival of *B. abortus* and that the protein also participates in maintenance of copper ion homeostasis in the macrophage host.

### ArsR2 affects expression of T4SS

**Figure 4.**
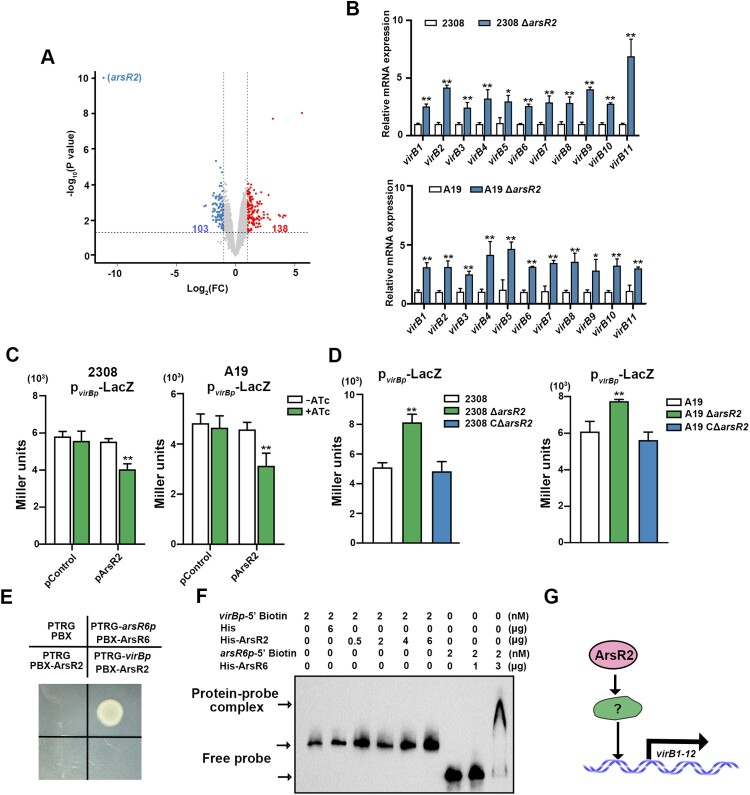
ArsR2 negatively regulates expression of T4SS products. (A) Volcano plot of differentially-expressed genes (Log_2_ FC > 1 and *P* value < 0.05) in *B. abortus* A19 and A19 Δ*arsR2*. The horizontal axis is the log_2_ FC between these strains. The negative log10 of the *P* value of Fisher’s exact test is plotted on the vertical axis. Each gene is represented by one point on the graph. (B) Schematic representation of *Brucella* T4SS encoded by the *virB* operon which comprises 12 open reading frames (*virB1* to *virB12*). (C) RNA-seq analysis of expression of *virB1*-*virB12* (Log_2_ FC > 1 and *P* value < 0.05) in *B. abortus* A19. (D) Expression of *virB1*-*virB12* was determined by qRT-PCR and normalized to gene expression in *B. abortus* S2308, A19 or deletion derivatives. (E) Activity of a plasmid-borne p*virBp*-*lacZ* transcriptional reporter in S2308 and A19 with or without ATc induction of ArsR2. Plasmids pControl and p*virBp*-*lacZ* or pArsR2 and p*virBp*-*lacZ* were electroporated into *B. abortus* S2308 and A19, respectively. The β-galactosidase activity was detected in the presence or absence of 200 ng/mL ATc. pControl indicates pBB-PZT1 empty vector and P*virBp* indicates pBB-PZT1-VirBp plasmid. Strains were grown in TSB to OD_600_ 0.6 in the absence or presence of 100 nM ATc. (F) Activity of a plasmid-borne p*virBp*-*lacZ* transcriptional reporter in *B. abortus* S2308, A19 strain or deletion derivatives. Relevant plasmids were electroporated into the required host strains and β-galactosidase activity was assessed in strains grown in TSB to OD_600_ 0.6. (G) B1H assays for the interaction between ArsR2 and *virB* promoter. *E. coli* XR reporter strains containing the pTRG, pBX plasmids and derivatives were cultured and spotted on LB agar in the presence or absence of streptomycin and 3-AT. The pTRG and pBX empty vectors were used as negative controls. The pBRG-ArsR6 and pBX-ArsR6 plasmids were used as positive controls and pTRG and pBX-ArsR2 were used as self-activation controls. (H) EMSA of His-ArsR2 binding to the *virB* promoter region. His-tag was used as a negative control and ArsR6 was incubated with *arsR6p* as a positive control. (I) Model for indirect regulation of *virB1-virB12* by ArsR2. Data shown in panels F-H are mean and standard deviation. *<0.05, **<0.01 by unpaired two-tailed t tests with Welch’s correction.

Comparative transcriptomic analysis using RNA-seq was performed for A19 and A19 Δ*arsR2* strains to assess whether ArsR2 affects global gene expression. A total of 241 genes were expressed differentially of which 138 genes were up-regulated and 103 genes were down-regulated in the deletion strain compared to wild-type (Figure 4(A)). Further analysis using Gene Ontology (GO) on differentially expressed genes highlighted ArsR2’s regulatory effects (Figure S4). T4SS is a crucial virulence factor that plays important roles in intracellular survival of *Brucella* and in regulating the host immune response to infection [8,26–28]. Analysis by qRT-PCR verified the changed expression of *virB1-11* in Δ*arsR2* compared with wild-type strain (Figure 4(B)). These data strongly suggest that ArsR2 participates in regulating expression of T4SS. The influence of ArsR2 on the expression of *virB1-11* was explored further using a *virB-lacZ* transcriptional fusion in *B. abortus*. Overexpression of ArsR2 decreased the activity of the fusion (Figure 4(C)). In contrast, expression of the *virB-lacZ* fusion was enhanced in Δ*arsR2* compared to wild-type and CΔ*arsR2* strains (Figure 4(D)), indicating that the *virB1-11* expression is repressed by ArsR2. To determine if ArsR2 regulation of T4SS is directed, B1H was performed to analyze the interaction between ArsR2 and *virB* promoter. Surprisingly, ArsR2 cannot bind to *virB* promoter region (Figure 4(E)). Consistent with this result, His-tagged ArsR2 protein did not recognize the *virB* promoter in EMSA (Figure 4(F)). Therefore, we conclude that expression of *virB1-11* that encodes the T4SS in *B. abortus* is regulated indirectly by ArsR2 (Figure 4(G)).

### ArsR2 regulated *vjbR* expression by binding the cognate promoter

**Figure 5.**
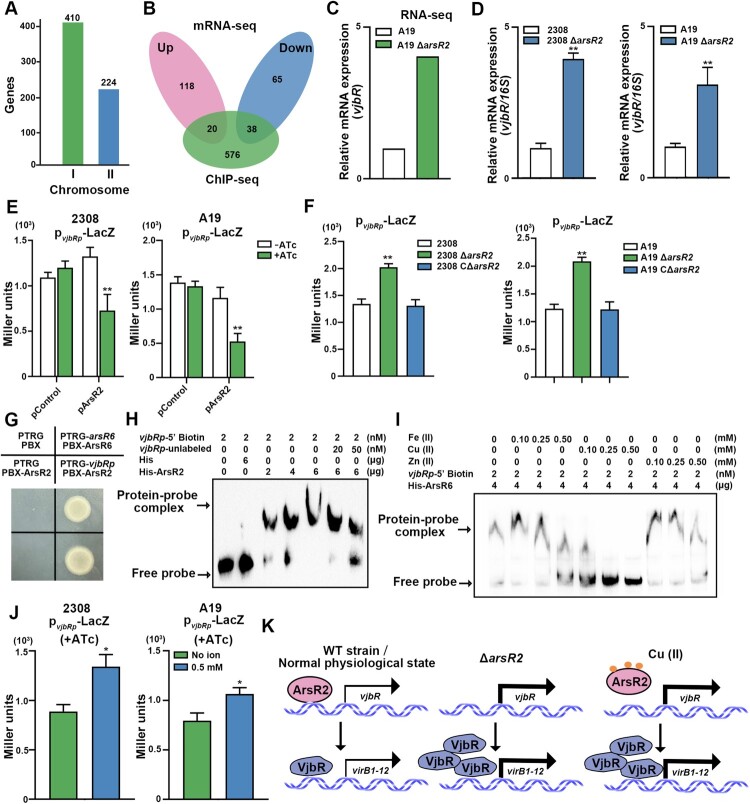
ArsR2 interacts with the *vjbR* promoter region and inhibits *vjbR* transcription. (A) ChIP-seq reveals potential target genes of ArsR2. *B. abortus* A19 CΔ*arsR2* in stationary phase was cross-linked, washed, and sonicated to produce sheared chromosomal DNA. DNA was purified were utilized for library construction to conduct ChIP-seq. (B) Analysis of RNA-Seq and ChIP-Seq data identifies the ArsR2 regulon. RNA-seq and ChIP-seq data were analyzed further to evaluate genes that potentially are regulated directly by ArsR2. The set of peak-associated genes was filtered using the list of differentially-expressed genes as determined by RNA-seq. The 58 differentially-expressed genes were identified as potential target genes for ArsR2 regulation. (C) Expression levels of were determined by qRT-PCR and normalized to gene expression in *B. abortus* S2308, A19 and derivatives. (D) Activity of the p*vjbR*-*lacZ* transcriptional reporter in S2308 and A19 with or without ATc. The pControl and p*vjbR*-*lacZ* plasmids or pArsR2 and p*vjbR*-*lacZ* were electroporated into *B. abortus* S2308 and A19, respectively, and β-galactosidase activity was detected in the presence or absence of 200 ng/mL ATc. pControl indicates pBB-PZT1 empty vector and P*vjbR* indicates pBB-PZT1-VjbR plasmid. Strains were grown in TSB to OD_600_ 0.6 in the absence or presence of 100 nM ATc. (E) Activity of the p*vjbR*-*lacZ* transcriptional reporter in *B. abortus* S2308, A19 and derivatives. β-galactosidase activity was assayed in strains grown in TSB to OD_600_ 0.6. (F) B1H assays for the interaction between ArsR2 and *vjbR* promoter. E. coli XR reporter strains containing the pTRG, pBX plasmids and derivatives were cultured and spotted on LB agar in the presence or absence of streptomycin and 3-AT. The pBRG and pBX empty vectors were used as a negative control, pBRG-ArsR6 and pBX-ArsR6 were used as a positive control, and pBRG and pBX-ArsR2 was used as a self-activation control. (G) EMSA of His-ArsR2 binding to the *vjbR* promoter. His-tag was incubated with the promoter as a negative control. Unlabelled *arsR2* promoter sequence was tested for competition with the biotin-labelled *vjbR* promoter. Results are representative of at least three independent experiments. (H) The effect of metal ions on DNA binding activity of ArsR2. The *arsR6* promoter region probe was incubated with ArsR6 (2 µg) in the presence or absence of metal ions. Results are representative of at least three independent experiments. (I) Activity of a plasmid-borne p*vjbR*-*lacZ* transcriptional reporter in *B. abortus* S2308 and A19 in the present or absence of copper ions. The p*vjbR*-LacZ plasmid was electroporated into the strains, and β-galactosidase activity was assayed in strains grown in TSB to OD_600_ 0.6. (J) Schematic depiction of the outcome of ArsR2 regulation of T4SS genes by VjbR in the wild-type strain, Δ*arsR2*, and in the presence of copper ions.

ChIP-Seq was performed to identify genomic binding sites for ArsR2 and to further characterize the ArsR2 regulon in *B. abortus*. ArsR2 matches 411 and 224 target genes on chromosomes I and II, respectively (Figure 5(A)). RNA-seq and ChIP-seq data were analyzed further to evaluate genes that potentially are regulated directly by ArsR2. The set of peak-associated genes was filtered using the list of differentially-expressed genes as determined by RNA-seq which revealed 58 differentially-expressed genes in the ChIP-seq data of which 20 genes were up-regulated and 38 genes were down-regulated (Figure 5(B)). Interestingly, ArsR2 bound upstream of *vjbR* which encodes a quorum sensing-dependent transcriptional regulator. The two-component regulator BvrRS allows *Brucella* to sense acidic pH and nutrient deprivation in eBCVs which activates expression of the quorum-sensing regulator VjbR which in turn promotes *virB* expression [29]. In addition, VjbR plays an essential role in virulence, because its deletion strain is highly attenuated [30,31]. Analysis by qRT-PCR showed that *vjbR* was up-regulated in the Δ*arsR2* background compared with wild type strain (Figure 5(C,D)). The influence of ArsR2 on expression of *vjbR* was explored further using a *vjbR-lacZ* fusion. We observed that the overexpression of ArsR2 inhibited *vjbR* promoter β-Galactosidase activity in *B. abortus* (Figure 5(E)). Compared to the wild type strain, the *vjbR* promoter β-Galactosidase activity was increased in Δ*arsR2* (Figure 5(F)). These results show that the *vjbR* expression was repressed by ArsR2. B1H assays were performed to analyze further the interaction between ArsR2 and the *vjbR* promoter and to determine if ArsR2 regulation of the promoter is direct. ArsR2 bound to the *virB* promoter region in these assays (Figure 5(G)). Consistent with this observation, purified His-tagged ArsR2 protein assembled on the *vjbR* promoter region in EMSA (Figure 5(H)). Thus, we conclude that expression of *vjbR* is repressed directly by ArsR2.

EMSA analysis was used to determine the effect of metal ions on ArsR2-*vjbRp* complex formation. We found that ArsR2-DNA complex was not dissociated in the present of Fe (III) (Figure 5(I)), suggesting that Fe (III) does not affect the binding of ArsR2 to the *vjbR* promoter. However, addition of 0.25 mM Cu (II) was sufficient to dissociate the ArsR2-*vjbRp* complex (Figure 5(I)). Thus, Cu (II) reduces the affinity of ArsR2 for the promoter region. Consistent with these results, overproduction of ArsR2 in the presence of exogenous Cu (II) enhanced *vjbR* promoter activity (Figure 5(J)). In summary, Cu (II) plays a crucial role in ArsR2-mediated regulation of *vjbR* as depicted in the model in Figure 5(K).

### Copper does not affect the regulation of T4SS products by VjbR

**Figure 6.**
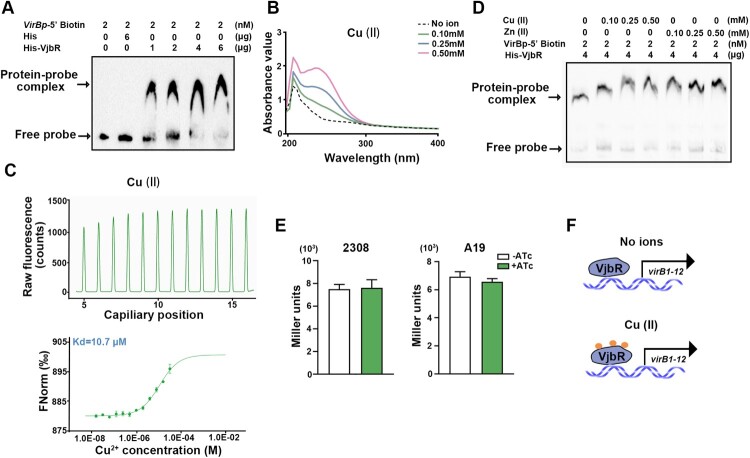
Copper ions do not impact the interaction between VjbR and *virB* promoter. (A) EMSA of His-VjbR and the *virB* promoter region. His-tag was incubated with the fragment as a negative control. Results are representative of at least three independent experiments. (B) Absorption spectra of VjbR (50 μg/mL) after incubation with different concentrations of Cu (II). (C) Interactions between ArsR2 protein and Cu (II) by MST. *K_d_* represents the dissociation constant. (D) The effect of Cu (II) on DNA binding activity of VjbR. The *virB* promoter region probe was incubated with VjbR (2 µg) in the presence or absence of Cu (II). Results are representative of at least three independent experiments.

Previous research has demonstrated that VjbR can bind to *virB* promoter and activate *virB* expression [7]. Consistent with these results, we observed that His-tagged VjbR protein bind to *virB* promoter in EMSA (Figure 6(A)). As Cu (II) plays an important role in regulating *vjbR* by ArsR2, the role of copper in controlling expression of T4SS by VjbR was assessed. Absorbance values increased significantly in the presence of Cu (II) which indicated that VjbR binds copper (Figure 6(B)). MST verified that VjbR bound to Cu (II) directly and specifically (Figure 6(C)) with *K_d_* = 10.7 μM (Figure 6(C)). As these findings demonstrate that VjbR binds Cu (II), the impact of copper on VjbR transcriptional activity was examined. The VjbR-*virB* promoter complex was not affected by Cu (II) (Figure 6(D)). Consistent with these results, activity of the *vjbR-lacZ* fusion in Δ*arsR2* was not significantly different in the present or absence of exogenous Cu (II) (Figure 6(E)). These combined data indicate that, although VjbR binds Cu (II) *in vitro*, copper does not affect VjbR expression as depicted in Figure 6(F).

### ArsR2 contributes to *B. abortus* virulence

**Figure 7.**
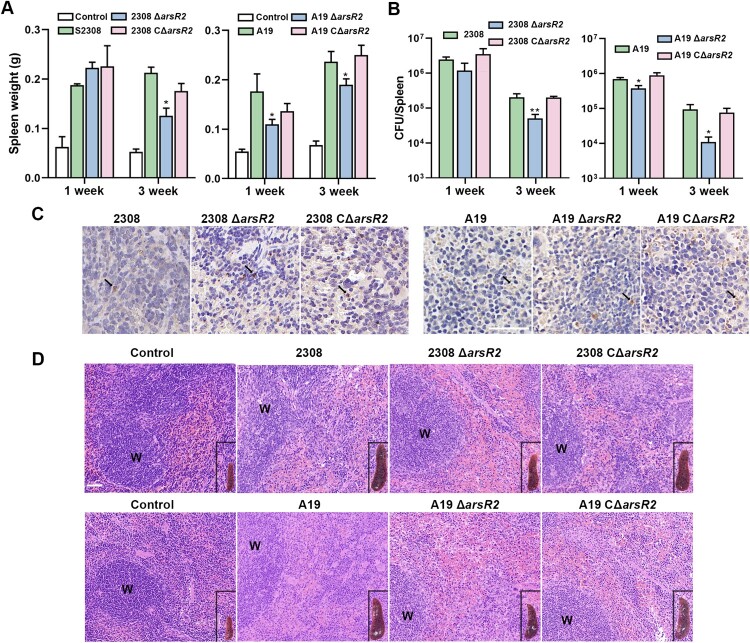
ArsR2 is crucial for *B. abortus* virulence. (A) The spleen weight of BALB/c mice infected with *B. abortus* or derivatives at one and four weeks post-infection. Data represent mean and standard deviation. *<0.05 by one-way analysis of variation followed by Tukey’s multiple-comparison test. (B) CFU of *B. abortus* and derivatives in spleens of infected mice at weeks one and four post-infection. Data represent mean and standard deviation. *<0.05 by one-way analysis of variation followed by Tukey’s multiple-comparison test. (C) Immunohistochemical staining for *B. abortus* in spleen sections of BALB/c mice infected with *B. abortus* or derivatives at four weeks post-infection. Arrows indicate examples of clusters of positive-stained cells. Scale bar represents 50 μm. (D) Representative micrographs of spleen histopathology at three weeks post-infection. Scale bar represents 50 μm. W, white pulp.

As the preceding data demonstrate a role for ArsR2 in copper ion homeostasis and regulation of T4SS via VjbR, we evaluated the impact of ArsR2 in *B. abortus* virulence using a BALB/c mice infection model. Four-to-six week old mice were infected with *B. abortus* S2308 or Δ*arsR2* deletion derivatives (1 × 10^5^ CFU) or *B. abortus* A19 or derivatives (1 × 10^7^ CFU) and bacterial survival in spleen was monitored up to three weeks post-infection. Mice infected with wild-type or CΔ*arsR2* strains had significantly increased spleen weights compared to uninfected animals, whereas spleen weight decreased in mice infected with the deletion strain (Figure 7(A)). Furthermore, a significant reduction in spleen bacterial burden was observed in mice infected with Δ*arsR2* compared to the wild-type at weeks one and three post-infection (Figure 7(B,C)), indicating that ArsR2 is required with *B. abortus* virulence in mice infection model. Histopathological analyses revealed that spleens infected with Δ*arsR2* exhibited slight white pulp expansion and a clearly visible boundary (Figure 7(D)). Together, these findings demonstrate that ArsR2 plays a pivotal role in regulating bacterial virulence in mice.

## Discussion

**Figure 8.**
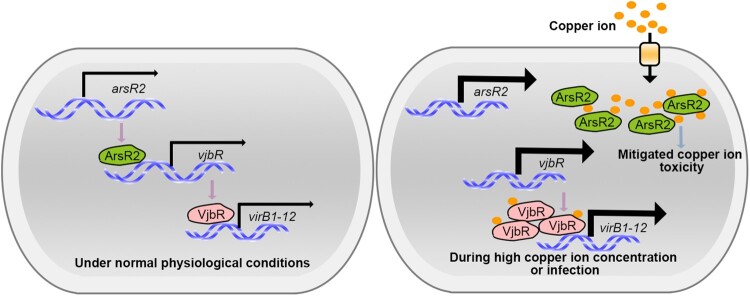
Model for the transcriptional regulation of T4SS by the copper-sensing transcription factor ArsR2. Under normal physiological conditions, ArsR2 inhibited T4SS expression through the LuxR-type Regulator VjbR. However, during high copper ion concentration or infection, ArsR2 mitigated copper ion toxicity. Furthermore, copper modulated the transcriptional activity of ArsR2, leading to an upregulation of T4SS expression, while VjbR remained unaffected.

Bacteria in the genus *Brucella* are significant human and veterinary pathogens. A comprehensive analysis of the pathogenesis of Brucella is essential for the development of novel vaccines against this infection. ArsR transcriptional regulators exert key roles in diverse crucial cellular processes, but little is known about the activity of these factors in *Brucella*. Here, we demonstrated that ArsR2 is an important regulator of metal ion homeostasis and virulence in *B. abortus*. Although lacking autoregulatory activity, ArsR2 is required for copper ion homeostasis and thus may act as a metalloregulatory factor that detoxifies environmental copper ions. In addition, a combination of RNA-seq and ChIP-seq revealed that ArsR2 is a repressor of the *vjbR* gene that regulates T4SS expression. Copper ions reduced the affinity of ArsR2 for the *vjbR* promoter thereby disinhibiting *vjbR* expression and further activating expression of T4SS. However, copper did not independently affect VjbR-mediated repression of the *virB* operon. ArsR2-deficient mutants showed severe attenuation in a mouse infection model. Thus, ArsR2 is involved in *Brucella* virulence by maintaining metal ion homeostasis and by regulating the crucial T4SS virulence complex via VjbR (Figure 8).

Multiple transcription factors involved in *Brucella* virulence have been identified, including GntR, LysR, and ArsR family members [32]. *Brucella* encodes six ArsR homologs, among which ArsR6 previously was implicated in virulence [33,34]. However, the functions of other ArsR proteins in *Brucella* remain uncertain. ArsR proteins bind to the cognate promoters to autoinhibit expression. ArsR2 was highly conserved in *Brucella* but does not bind the cognate promoter which indicates that the protein is an atypical ArsR family member. As ArsR factors maintain metal ion homeostasis under extreme environmental conditions, including in *Mycobacteriun tuberculosis* [35,36], we explored whether ArsR2 exerts a role in metal ion balance in *Brucella*. Indeed, ArsR2 possesses high copper-chelating activity and participates in copper ion homeostasis in *B. abortus* both *in vitro* and in macrophages which prompted us to further characterize the ArsR2 regulon.

T4SS is an essential virulence factor that is implicated in bacterial intracellular survival and manipulating host immune response to *Brucella* infection. The complex is encoded by the *virB* operon that is regulated by the promoter upstream of *virB1* [37,38]. Strains with *virB* deletions are stalled in eBCVs and unable to convert eBCVs into rBCVs to facilitate *Brucella* replication indicating that T4SS is required for rBCVs biogenesis [8,9,28]. All subunits of T4SS are crucial for virulence, apart from VirB1, VirB7, and VirB12 [8,28]. We showed here that ArsR2 modulated the expression of T4SS, but that this effect is not mediated directly by repression of *virB* operon transcription. Future work will be geared towards understanding the mechanism that underpins ArsR2-mediated control of T4SS.

It is not surprising that T4SS transcription is controlled by numerous regulators, including the two-component regulator BvrRS, quorum-sensing regulator VjbR, and stringent response regulator Rsh [39,40]. However, it is unknown how these regulators act in concert to dictate appropriate expression of T4SS in different conditions. VjbR, a LuxR-type family transcriptional regulator, play an important role in *Brucella* virulence, because it directly controls the expression of the T4SS. Combining RNA-seq and ChIP-Seq revealed that ArsR2 inhibited expression of VjbR which further highlights the significance of ArsR2 in *Brucella* infection. Metal ions may affect transcription factor binding to promoters thereby altering transcriptional activity [41–43]. Accordingly, copper induced dissociation of the ArsR2-*vjbR* promoter complex which led to enhanced expression of *vjbR*. However, copper independently did not perturb expression of T4SS via VjbR.

The accumulation of copper ions within macrophage phagolysosomes at infection sites contributes to bacterial killing through multiple mechanisms [44,45]. Thus, copper resistance is necessary for bacterial virulence, including in *Mycobacterium tuberculosis* [46]. The ArsR2 protein can be added to the catalog of factors that mediate copper homeostasis. The protein not only plays a vital role in controlling expression of T4SS via VjbR, but also was essential for *Brucella* virulence in the BALB/c mouse model. While the inhibition of VjbR by ArsR2 may seem contradictory to the decreased virulence of arsR2 deleted strains, this is not the case. When *Brucella* encounters copper ions inside the cells, ArsR2 serves as a copper-binding protein, reducing copper ion toxicity; at the same time, ArsR2’s reduced ability to bind vjbR promoters after binding with copper ions, results in increased vjbR expression, aiding Brucella’s intracellular survival. However, after the deletion of ArsR2, although T4SS was activated through vjbR, the ability to reduce copper ion toxicity is lost. This imbalance leads to a decrease in bacterial virulence. In conclusion, we have uncovered the function of ArsR2 in metal ion homeostasis and in regulating crucial virulence genes and explored the impact of the protein on *Brucella* virulence. Based on these observations, ArsR2 is a potentially attractive target for chemotherapeutic intervention and as an attenuated live vaccine to combat *Brucella* infection.

## Methods

### Bacterial strains, culture conditions, and reagents

Experiments were performed using *B. abortus* S2308 (CVCC788), *B. abortus* A19 (CVCC70202) or its derivatives. When is appropriate, kanamycin (50 μg/ml), carbenicillin (50 μg/ml), gentamycin (50 μg/ml) or ATc (200 ng/ml) were added. Experiments using live *B. abortus* 2308 were performed in Biosafety Level 3 facilities at the Chinese Academy of Agricultural Sciences, China. *Escherichia coli* DH5α (TIANGEN, CB101) and *E. coli* BL21 (DE3) (TIANGEN, CB105) were used for cloning and protein expression, respectively. *E. coli* XL1-Blue MRF´ Kan strains for one-hybrid studies were derived from the BacterioMatch@II Two-Hybrid System (Stratagene, #240065). *B. abortus* strains were cultured in TSB to OD_600_ 0.6 for subsequent experiments.

### Construction of *Brucella* deletion and complementation strains

The *arsR2* deletion of *B. abortus* S2308 was generated via allelic replacement. Briefly, upstream and downstream fragments of *arsR2* and the kanamycin-resistance gene were amplified. The products were used as templates for overlap PCR. Purified PCR product was cloned into pMD-19T via the DNA Ligation Kit (TaKaRa, 6022). Recombinant plasmids were verified by PCR and sequencing and were electroporated into competent *B. abortus* cells. The deletion strain was selected on TSA containing kanamycin, and was confirmed by PCR, and sequencing. The *arsR2* deletion strains of *B. abortus* A19 were generated via double homologous recombination. Briefly, upstream and downstream fragments of the target genes were amplified. The products were used as templates for overlap PCR. PCR products were cloned into HindIII-digested pSC via the ClonExpress II One Step Cloning kit (Vazyme, C112-01) and clones were verified by PCR and sequencing. The resulting suicide plasmids were electroporated into competent *B. abortus*. The first homologous recombination was selected on TSA containing kanamycin and the second recombination was selected on TSA containing sucrose (8%). Deletion strains were verified by PCR and sequencing. Deletion strains were complemented by reintroducing the relevant gene and promoter region using the pBBR1MCS shuttle plasmid [47]. Briefly, the gene and promoter and Flag-tag were amplified, cloned into pBBR1MCS, and verified by PCR and sequencing. The recombinant plasmid was electroporated into competent cells of the deletion strain. The complemented strain was selected on TSA containing kanamycin and confirmed by Western blot. Amplification primers are listed in Table S1.

### Protein cloning, expression and purification

*Brucella arsR2 or vjbR* was amplified, cloned into *EcoR* I-digested pET-32a plasmid via clonexpress II one step cloning kit, and verified by PCR and sequencing. The recombinant plasmid was transformed into competent *E. coli* BL21 (DE3) cells for protein expression. Cells were collected by centrifugation and resuspended in cold lysis buffer, and lysed using JN-02C high pressure homogenizer. The supernatant was collected after centrifugation and His-tagged protein was purified by nickel affinity chromatography using the His-tag Protein Purification Kit (Beyotime, P2226) followed by size exclusion chromatography. Purified protein was digested with enterokinase (Solarbio, E8350) to remove the TrxA-6×His-thrombin-S tag, if required. For ultraviolet light assays, protein was placed in a quartz cuvette, different metal ion concentrations were added, and absorption spectra were detected by spectrophotometry.

### MicroScale thermophoresis assay

Protein affinity for metal ions was measured by MST. Proteins were labelled with Protein Labelling Kit RED-NHS 2nd Generation Kit (Monolith, MO-L011) and were used in MST assays at 40 nM. For titration assays, metal ion concentrations from 0.5 mM–15.3 nM (two times gradient dilution) were transferred to protein solutions in 100 mM Bis-Tris, 0.05% Tween-20, 150 mM NaCl, pH 8. Samples were incubated for 20 min at room temperature and then were filled into premium capillaries. Measurements were taken on a Monolith NT.115 MST system and MO affinity analysis software v2.3 was used for data analysis. Equilibrium dissociation constant (*K_d_*) values were obtained using Fnorm (‰).

### Western blot

Lysates were separated on SDS-PAGE and transferred to PVDF membranes. Membranes were blocked with 5% skim milk and incubated with primary Flag antibody (TransGen Biotech, HT201). Blots were incubated with anti-mouse IgG secondary antibody and were visualized using a gel image system.

### qRT-PCR

*B. abortus* strains were cultured in TSB to OD_600_ 0.6. Cells were collected and total RNA was extracted using RNAprep Pure Cell/Bacteria Kit. Reverse transcription was performed using the RT Reagent Kit (Vazyme, R333) and RT–PCR was performed using ChamQ SYBR qPCR Master Mix (Vazyme, Q311) on ABI 7500 system. Data analysis used the 2^−ΔΔCt^ method. Primers for qRT-PCR are listed in Table S1.

### Bacterial one-hybrid analysis

The interaction between ArsR2 and promoter of the target gene was detected using B1H analysis as described previously [48]. Briefly, the promoter was cloned into the pBX plasmid and the *arsR* gene was cloned into the pTRG vector. The empty vectors served as negative controls. The pBX-ArsR6 and pTRG-ArsR6 plasmids were used as positive controls and pBX-ArsR2 and empty vector pTRG served as the self-activation control. Selective agar medium contained streptomycin (16 μg/ml), 3-AT (20 mM), tetracycline (15 μg/ml), chloramphenicol (34 μg/ml), and kanamycin (50 μg/ml).

### Electrophoretic mobility shift assay

Fragments were amplified from *B. abortus* genomic DNA using appropriate primers (Table S1). DNA probes were labelled with biotin using the EMSA Probe Biotin Labelling Kit (Beyotime, GS008) and were mixed with different protein concentrations in gel-shift buffer. Reactions were incubated at room temperature for 20 min and were resolved by PAGE (5%) in 0.5X TBE buffer. DNA was detected using the Chemiluminescent Biotin-Labelled Nucleic Acid Detection Kit (Beyotime, GS009).

### β-galactosidase activity assay

A series of *lacZ* fusion plasmids was generated and transformed into *B. abortus* and derivatives to obtain reporter strains. β-galactosidase activity was measure as described previously [25].

### RNA-seq experiment

*B. abortus* A19 and Δ*arsR2* cultures in logarithmic growth phase were collected for total RNA extraction. Libraries were sequenced using a Hiseq2500 sequencer. Raw sequencing reads were cleaned by removing adaptor sequences, reads containing poly-N sequences, and low-quality reads, and clean reads were mapped to the reference genome using HISAT40. Normalization was performed and fragments per kilobase per million mapped reads were calculated using RESM software. Adjusted *P* value ≤ 0.05 and absolute value of log2 Ratio ≥1 were used to identify differentially expressed genes. The RNA-seq data are deposited in the Sequence Read Archive (SRA) repository, accession number PRJNA1112605.

### ChIP-seq experiment

*B. abortus* A19 CΔ*arsR2* in logarithmic growth phase was collected, fixed with paraformaldehyde (1%), and sonicated on ice to shear DNA to approximately 250 bp. Fragments were immunoprecipitated with anti-Flag antibody (TransGen Biotech, HT201). Reverse crosslinking was conducted and DNA was extracted, resuspended, and assayed using the Illumina HiSeq sequenator. The ChIP-seq data are deposited in the Sequence Read Archive (SRA) repository, accession number PRJNA1112975.

### Macrophage infection

Murine macrophage RAW 264.7 cells were seeded in 24-well plates (2 × 10^5^ cells per well) and infected with *B. abortus* A19 and derivatives for 4 h or *B. abortus* S2308 and derivatives for 1 h at multiplicity of infection of 200:1. Cells were incubated for 1 h with cell culture medium containing 50 µg/mL gentamicin to eliminate extracellular bacteria and then in medium containing gentamicin (25 µg/mL) to avert continuous infection. RAW 264.7 cells were lysed in 0.5 mL of PBS-0.5% Triton X-100 and the lysates were plated on TSA to determine CFUs. For immunofluorescence assay, RAW 264.7 cells were fixed with paraformaldehyde (4%) and blocked with Immunol Staining Blocking Buffer. Triton X-100 (0.25%) was used to increase cell membrane permeability. Mouse anti-*Brucella* polyclonal antibody and rabbit anti-Lamp1 antibody (Abcam, ab24170) were used as primary antibodies and donkey anti-mouse Alexa Fluor 555 (Beyotime, A0460) and goat anti-rabbit Alexa Fluor 555 (Beyotime, A0423) were used as secondary antibodies. Subsequently, coverslips were mounted on glass slides, and the cells were observed by microscope.

### Mouse infection

Six-to-eight week old BALB/c female mice were infected intraperitoneally with approximately 10^7^ CFU *B. abortus* A19 and derivatives or 10^5^ CFU *B. abortus* S2308 and derivatives. Bacterial loads in spleen were enumerated on TSA agar. Spleens were weighed for evaluation of splenomegaly and stained by H&E for examination of pathological features. All animal experiments were approved by t the Committee for the Ethics of Animal Experiments of the Lanzhou Veterinary Research Institute at the Chinese Academy of Agricultural Sciences (LVRIAEC-2023-066).

### Statistical analysis

Experiments were performed as three independent biological replicates. SPSS 22 software was used for statistical comparison. Results are presented as means ± standard deviation. Further analyses were performed using unpaired two-tailed t tests with Welch’s correction and one-way analysis of variation followed by Tukey’s multiple-comparison test. Probability (*P*) values < 0.05 were considered statistically.

## Supplementary Material

Figure S2.tif

Figure S3.tif

Figure S4.tif

Figure S1.tif

Table S1 Oligonucleotides used in the study.docx
