# Supplementary figures and images for: Copper sensing transcription factor ArsR2 regulates VjbR to sustain virulence in Brucella abortus
Source: Emerg Microbes Infect. 2024 Sep 19;13(1):2406274. doi: 10.1080/22221751.2024.2406274 (PMC11425708; doi:10.1080/22221751.2024.2406274)

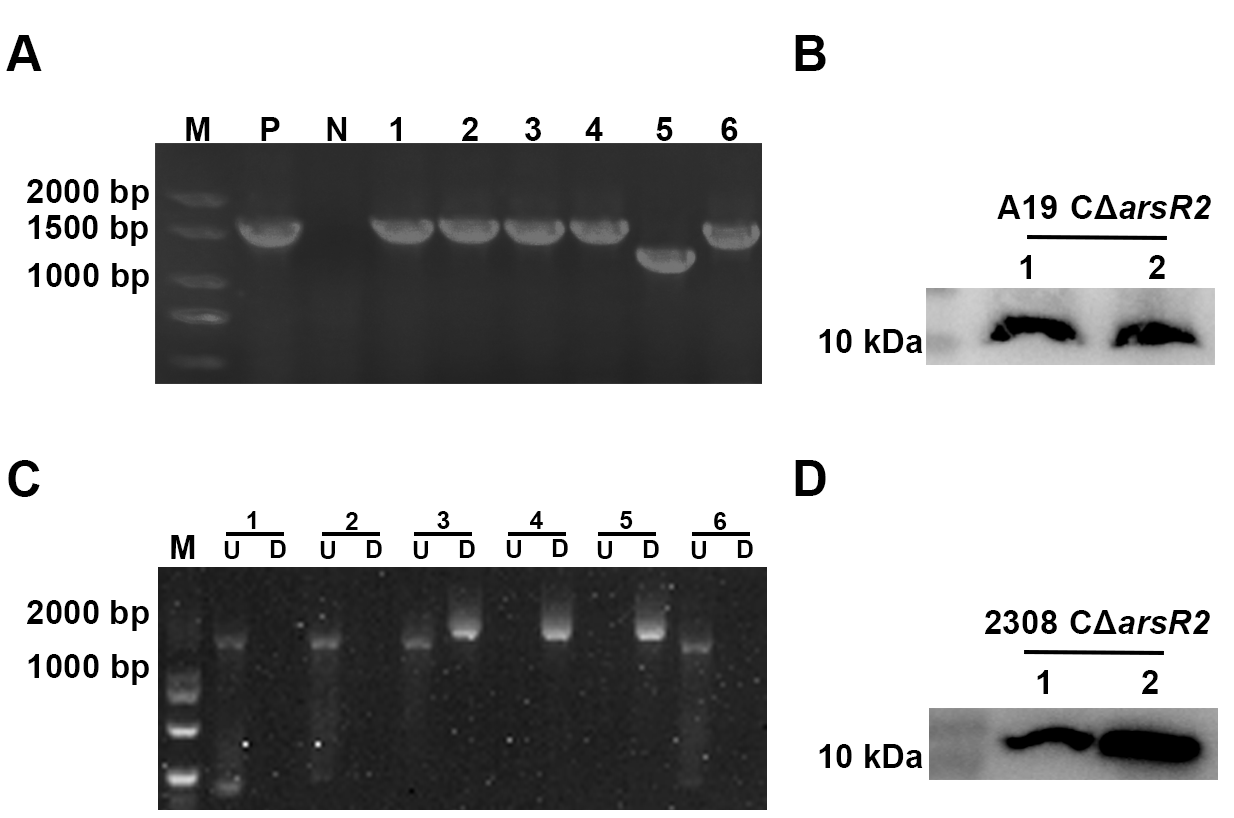

Supplement: Figure S2.tif [file TEMI_A_2406274_SM2308.tif]

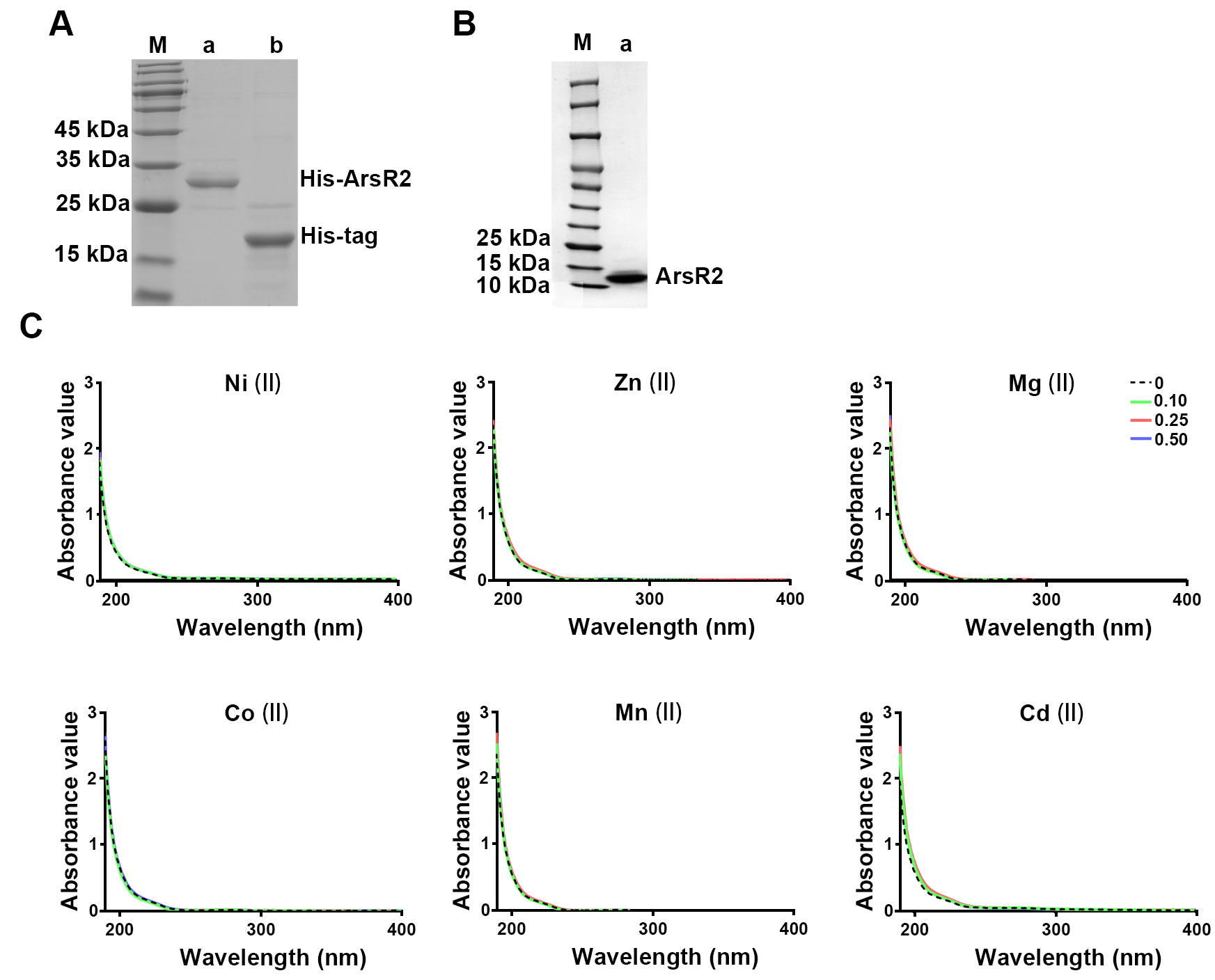

Supplement: Figure S3.tif [file TEMI_A_2406274_SM2307.tif]

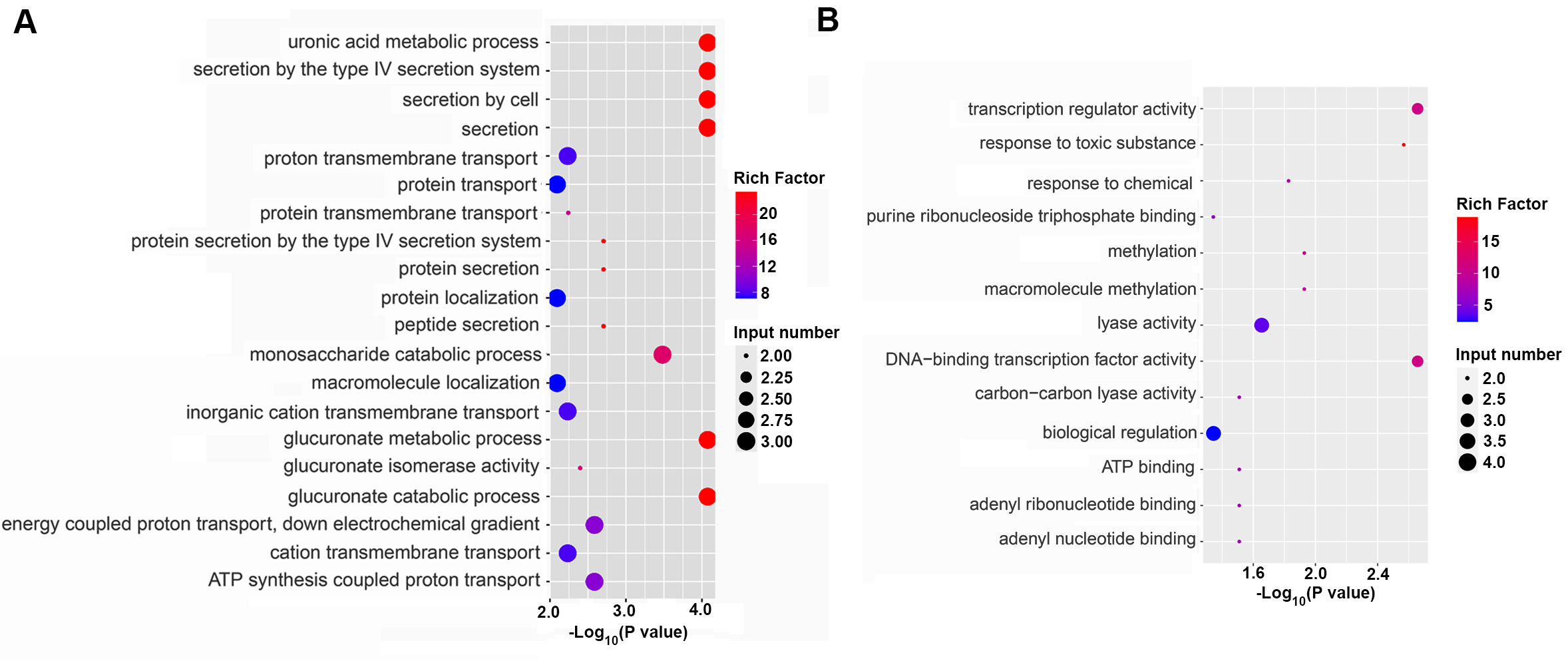

Supplement: Figure S4.tif [file TEMI_A_2406274_SM2306.tif]

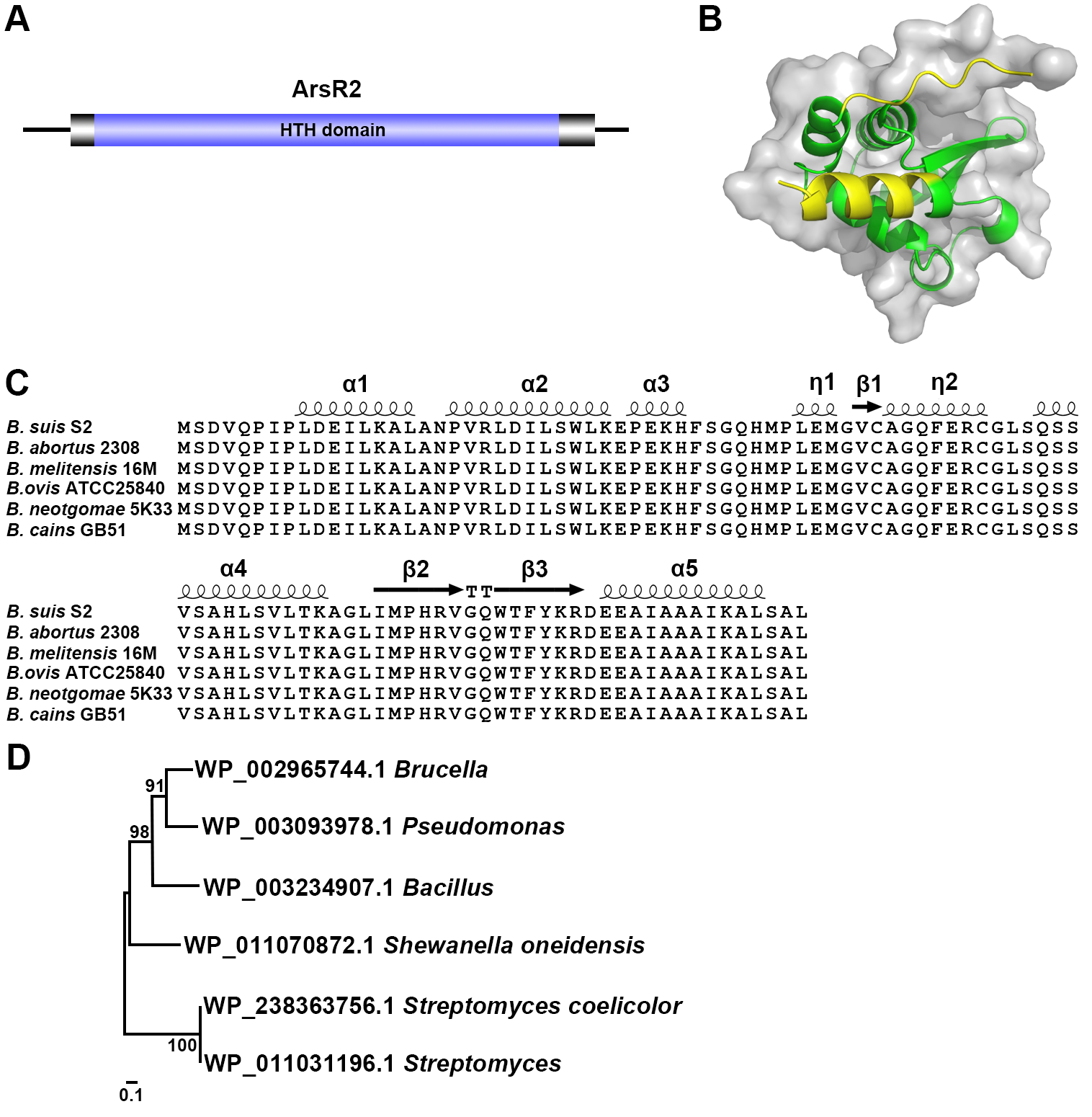

Supplement: Figure S1.tif [file TEMI_A_2406274_SM2305.tif]
