# Supplementary material for: Copper sensing transcription factor ArsR2 regulates VjbR to sustain virulence in Brucella abortus
Source: Emerg Microbes Infect. 2024 Sep 19;13(1):2406274. doi: 10.1080/22221751.2024.2406274 (PMC11425708; doi:10.1080/22221751.2024.2406274)
Supplement: Table S1 Oligonucleotides used in the study.docx [file TEMI_A_2406274_SM2304.docx]

**Table S1 Oligonucleotides used in the study**

| Name | Primer sequences | Usage |
| --- | --- | --- |
| ArsR2-Up-F | ACCTGCAGGCATGCAAGCTTTTGCAATCTTGAGCGCCAG | To amplify *arsR2* upstream fragment (A19) |
| ArsR2-Up-R | AAGATCGGTTTCAGGACGGGTGCAGTCACCTCGCCAGT |  |
| ArsR2-Down-F | ACACTGGCGAGGTGACTGCACCCGTCCTGAAACCGATCTT | To amplify *arsR2* downstream fragment (A19) |
| ArsR2-Down-R | ACCATGATTACGCCAAGCTTCACGGCATCCACAACTTCAAACAGA |  |
| Δ*arsR2*-F | TTCCAGTTCCGCCTTGGTTT | Used for Δ*arsR2* selection and verification |
| Δ*arsR2*-R | CAACTTTTCCGGCCCCAATG |  |
| cΔ*arsR2*-F | TTTTATCAGGCTCTGGGAGGGTAGTTTCCAATTCTCCACTATCAC | Used for CΔ*arsR2* construction |
| cΔ*arsR2*-R | CAAGAACTCTGTAGCACCGCTTACTTGTCATCGTCGTCCTTGTAATCAAGAGCAGACAGGGCTTTGATG |  |
| pET32a-ArsR2-F | CTGATATCGGATCCGAATTCATGTCTGATGTTCAGCCTAT | To amplify ArsR2 coding region |
| pET32a-ArsR2-R | TGTCGACGGAGCTCGAATTCAAGAGCAGACAGGGCTTTGA |  |
| pET32a-VjbR-F | CTGATATCGGATCCGAATTCATGAGTCTTGATCTCGTTCA | To amplify ArsR2 coding region |
| pET32a-VjbR-R | TGTCGACGGAGCTCGAATTCGACGAGATGCTGTACCTC |  |
| RT-VirB1-F | TGCCATTCCTTGTCCTCG | Used for qRT-PCR |
| RT-VirB1-R | AAGGGAAACGGGTTGACG |  |
| RT-VirB2-F | CGCTGCAATCGAGCCTAA |  |
| RT-VirB2-R | GTGCCGGAATGCCATCTT |  |
| RT-VirB3-F | ATGACAAGCGGGATCACAGGA |  |
| RT-VirB3-R | ACAGGAATCCAACGCACGAAG |  |
| RT-VirB4-F | TCAAACAATGTGGCGCTGTG |  |
| RT-VirB4-R | CCACGTTGAACAACCTGCTG |  |
| RT-VirB5-F | GCTCATCACCGCTTCGTAGA |  |
| RT-VirB5-R | GGCCAACCATCTCGAACAGA |  |
| RT-VirB6-F | ATATCAGCACCTATTCGGAGTG |  |
| RT-VirB6-R | ATGGCTGCGATGTTCCAC |  |
| RT-VirB7-F | GTCAACGGGGTTAGGCTGAG |  |
| RT-VirB7-R | TCCTTGCCGGTTGCACTAC |  |
| RT-VirB8-F | AACGCGATAGCTCGTGACAT |  |
| RT-VirB8-R | TTTGCGAAGACGACAAAGCG |  |
| RT-VirB9-F | TGTGGCTCGCCTTGAACTCCC |  |
| RT-VirB9-R | CCCTCCTGGGCAACCAAAATC |  |
| RT-VirB10-F | GTCTGCACCATCGTCTTGTCT |  |
| RT-VirB10-R | CTCTTTGTCGTGGGCTTCATC |  |
| RT-VirB11-F | CAAGCCGTCTTTCACCCG |  |
| RT-VirB11-R | CTTCAATGCGAGCAGTTCC |  |
| RT-16S-F | ACGTGCTACAATGGTGGTGA |  |
| RT-16S-R | CAGAGTGCAATCCGAACTGA |  |
| RT-VjbR-F | AGCGATACTTTGGCGCTTCT |  |
| RT-VjbR-R | ATTGCGGTAATACGGAGCGT |  |
| RT-ArsR2-F | TGAAGGAGCCGGAGAAACAC |  |
| RT-ArsR2-R | AATCAACCCCGCCTTCGTC |  |
| LacZ-ArsR2-F | aaaataaacaaataggggttccgcgGTAGTTTCCAATTCTCCACTATCAC | Used for β-Galactosidase activity assay |
| LacZ-ArsR2-R | gttgtaaaacgacgggatctatcatTGCAGTCACCTCGCCAGT |  |
| LacZ-VjbR-F | aaaataaacaaataggggttccgcgCTTGCAGCACGCTTTTACGA |  |
| LacZ-VjbR-R | gttgtaaaacgacgggatctatcatTGGAAATATCCTTGGTGATGAAACC |  |
| **LacZ-T4SS-F** | aaaataaacaaataggggttccgcgTGCCGCCTTGTTCACCGG |  |
| **LacZ-T4SS-R** | gttgtaaaacgacgggatctatcatAGGATCGTCTCCTTCTCAGA |  |
| *arsR2p*-F | TGCAGTCACCTCGCCAGTGT | Used for EMSA |
| *arsR2p*-R | GTGATAGTGGAGAATTGGAAACTAC |  |
| *vjbRp*-F | GCAGTCATGCCATCATGG |  |
| *vjbRp*-R | TGGAAATATCCTTGGTGATGAAACC |  |
| *virBp*-F | AGGATCGTCTCCTTCTCAGAGAATG |  |
| *virBp*-R | TGCCGCCTTGTTCACCGG |  |
